# Sex differences in risk-taking and associative learning in rats

**DOI:** 10.1098/rsos.150485

**Published:** 2015-11-04

**Authors:** Jolle Wolter Jolles, Neeltje J. Boogert, Ruud van den Bos

**Affiliations:** 1 Department of Zoology, University of Cambridge, Downing Street, Cambridge CB2 3EJ, UK; 2 Faculty of Science, Department of Organismal Animal Physiology, Radboud University Nijmegen, Nijmegen, The Netherlands

**Keywords:** conditioning, exploratory behaviour, learning, rats, risk-taking, sex differences

## Abstract

In many species, males tend to have lower parental investment than females and greater variance in their reproductive success. Males might therefore be expected to adopt more high-risk, high-return behaviours than females. Next to risk-taking behaviour itself, sexes might also differ in how they respond to information and learn new associations owing to the fundamental link of these cognitive processes with the risk–reward axis. Here we investigated sex differences in both risk-taking and learned responses to risk by measuring male and female rats’ (*Rattus norvegicus*) behaviour across three contexts in an open field test containing cover. We found that when the environment was novel, males spent more time out of cover than females. Males also hid less when exposed to the test arena containing predator odour. By contrast, females explored more than males when the predator odour was removed (associatively learned risk). These results suggest that males are more risk-prone but behave more in line with previous experiences, while females are more risk-averse and more responsive to changes in their current environment. Our results suggest that male and female rats differ in how they cope with risk and highlight that a general link may exist between risk-taking behaviour and learning style.

## 1. Introduction

Risk plays a key role in the way animals cope with their environment. Individuals have to continuously trade-off risk-prone behaviours such as feeding with risk-averse ones such as vigilance [1,2]. How much risk individuals take is a strong determinant of their survival and reproductive success [3], and can thus have large fitness consequences [4]. Importantly, systematic differences in how individuals trade-off risks and rewards may arise because of variability in life-history strategies and resulting fitness expectations [5]. As males of many species tend to have lower parental investment and greater variance in reproductive success than females [6], sexual selection is expected to result in systematic differences in how males and females trade-off risks and rewards: males may be more inclined to adopt high-risk but potentially high-return behaviours, while females may be more likely to behave in ways that ensure their safety [6–8].

An increasing amount of research is being devoted to investigate sex differences in risk-taking [7,9–19]. Most studies are in line with expectations based on the mating system of the study species [7,9,10,20–23], such as in sticklebacks and guppies, where males’ reproductive success is more variable, and males are bolder than females [7,10]. However, other studies report no significant sex effects [12,24,25] or even show that the opposite sex takes more risk [19,26–31]. This lack of consensus regarding systematic sex differences in risk-related behaviour may partly be explained by the fact that a large range of different tests has been used—such as open field tests, novel environments, novel object tests and predatory threat—and that sex effects are often not consistent between tests [19,32]. Importantly, this suggests that different tests may actually measure different aspects of behaviour, or lack ecological validity, both resulting in discrepancies in their interpretation [19,33–35].

In addition to sex differences in the willingness to take risks, sex differences in life-history trade-offs may also affect how males and females process and use information, and thus affect their learning responses. Similar to the consequences of being bold, being faster at gathering information and making more rapid decisions may bring in more rewards, such as food or mates, but at the cost of increased predation risk [2,36]. By contrast, making decisions based on a slower, more detailed acquisition and assessment of information might be safer as it may help reduce uncertainty regarding predation risk and lower the variability of rewards [37]. Males may therefore be especially likely to be proactive and internally driven, which enables them to come in contact with to-be-learned stimuli faster and establish routines more quickly. By contrast, females may generally sample more and be more reactive, providing them with more detailed information about their environment and making them more flexible learners. Sex differences in learning and information processing have been studied primarily from a neuro-scientific and biomedical perspective [19,31,38,39]. In general, the findings are in line with our predictions: while males tend to be faster at making decisions, learn more quickly, and tend to show more perseverance [31,38,40–44], females tend to engage in a more reactive way with their environment [43,45,46], thereby acquiring more detailed and up-to-date information [39,41,44], and often retain associations more effectively [31].

Despite the considerable amount of research that has been devoted to sex differences in risk-taking, learning and decision-making by both behavioural ecologists and biomedical researchers [14,16,19,33,38,39,47], there is still little integration of research ideas and findings between them. Furthermore, cognition studies consider that memory and learning differences are often linked to the risk–reward axis [36,48,49]. To explicitly test the hypothesis that males are more inclined to adopt high-risk, high-return behaviours than females, here we investigate sex differences in both risk-taking behaviour *and* learned responses to cope with risk. We repeatedly subjected male and female Wistar rats (*Rattus norvegicus*) to an open field test and observed their behavioural responses when: (i) the environment was novel (i.e. during the first exposure), (ii) when the environment contained the odour of a predator (potentially high risk), both to look at risk-taking behaviour, and (iii) when the odour of the predator was removed, to quantify responses to associatively learned risk [47]. A hide-box was offered as rats are fossorial and naturally respond to threat by hiding or running away [36,50]. Here we use cat odour as an ecologically relevant alternative to the shock-based learning paradigms commonly used in biomedical research focused on sex differences in learning [19,38]: rats show strong defensive behaviours when exposed to the odour of a cat [47,51–53] as well as rapid context and cue conditioning to stimuli associated with the odour [53,54]. Our three-session experimental paradigm is based on the large body of experimental work focused on investigating effects of cat odour on rat behaviour (reviewed by [51,53,55,56]).

Rats have a polygynous to polygynandrous mating system [57] with females showing considerably higher parental investment [58], while males disperse, are territorial and compete with each other over access to burrows and females [57,59]. Based on these different life-history priorities, we expected that male rats would adopt more high-risk but potentially high-return behaviours, while females should be more sensitive to changes in their environment. We therefore hypothesized that males would spend less time hiding than females in the novel open field test and when exposed to the cat odour. By contrast, we expected females to be more sensitive and thus responsive to the removal of the predator odour, and therefore to spend more time out of cover in the conditioned-risk context.

## 2. Methods

### 2.1 Subjects and housing

Ten-week old male (*n*=30) and female (*n*=30) Wistar rats (*Rattus norvegicus*) acquired from Harlan (Horst, The Netherlands) were used as subjects. We housed the animals in temperature- and climate-controlled rooms (23±2°C, 45–65% humidity) with a reversed light–dark cycle (lights on from 19.00 to 07.00 h). During the dark cycle, red ceiling lights provided illumination. Background noise was provided by a radio playing top-40 music 24 h a day, 7 days a week. Rats were housed in same-sex pairs under enriched conditions, i.e. in Perspex Macrolon type IV cages that contained sawdust bedding and cardboard and tissues for enrichment and were covered by a metal grid (movement area: *ca* 35×38 cm, 17 cm height). Rat chow (Special Diets Services, Witham, Essex, England) and water were available ad libitum. Rats were allowed to habituate to the laboratory environment for seven weeks before testing, during which they were handled individually two to three times a week for 5 min. Males (average weight: 334.5 g, range: 316–355 g) and females (average weight: 230.5 g, range: 216–245 g) were housed in separate rooms and tested at 17 weeks of age in different weeks to avoid inter-sexual interference. Throughout the experimental period, we did not observe any aggressive interactions between pair-housed individuals nor injuries.

### 2.2 Experimental set-up

To investigate the rats’ behavioural responses to risk, we subjected them repeatedly to one of three identical open field tests that contained cover, conforming to other studies investigating the role of cat odour (reviewed by [47,53,55]). The open field consisted of a black acrylic surface area (450 mm width×450 mm length) with transparent acrylic walls (550 mm height; Noldus Phenotyper, Noldus Information Technology, Wageningen, The Netherlands; electronic supplementary material, figure S1). One corner of the arena contained a black acrylic box termed the ‘hide-box’ (14×14×12 cm), which contained a round opening (4 cm radius) 2.5 cm above the floor. The opposite corner of the arena contained an alligator clip 4 cm above the floor that held a piece of cotton towel fabric (3×15 cm strip fold-up to ±4 cm^2^), termed the ‘stimulus’. We used either a ‘cat odour stimulus’, created by placing the towel in laboratory cat beds for three weeks (see ethical note), or a ‘control stimulus’ that had not been in contact with a cat. Both control and cat odour stimuli were stored separately in airtight plastic containers in a freezer at −10°C and were always handled with plastic gloves. The rats’ movements were recorded using a camera in the centre of the top-unit of the apparatus.

### 2.3 Experimental procedure

During the dark phase, rats were subjected to the open field set-up for three 20 min sessions on three subsequent days [53,60]. On day 1, the arena contained the control stimulus and was novel to the rats (‘novel context’). On day 2 the arena, now not novel anymore, contained the cat odour stimulus (‘predator odour context’). On day 3, the arena contained the control stimulus again, thus serving as a ‘conditioned context’. We used three identical arenas to test the 30 rats of each sex in a total of 10 sessions each day. Rats were placed in the centre of the apparatus at the beginning of each trial. The testing arena and testing order were randomized each day. To avoid the fading of the cat odour, both the control and cat odour stimuli were changed using latex disposable gloves before testing a new subject. To avoid the transfer of rat odours, each arena was thoroughly cleaned with ethanol solution and paper towels after each trial. Between test days the arenas were thoroughly cleaned an extra time and dismantled until the next day.

### 2.4 Behavioural measures

Videos were analysed using Ethovision 3.1 (Noldus Information Technology, Wageningen, The Netherlands). Conforming to previous studies [52,54] we recorded the duration rats were completely hidden inside the hide-box (‘hidden’), with their body in the hide-box but with their head or head and shoulders outside the entrance, a characteristic risk-assessment posture [47] (‘head-out’), and with their body completely outside the hide-box (‘out’). For simplicity, we refer to rats spending time outside the hide-box as ‘exploring’. All recorded behaviours were checked afterwards for any inconsistencies. Note that these measures are mutually exclusive and reflect levels of risk-taking and/or engagement with the environment: low (hidden), intermediate (head-out) and high (out). We additionally measured a rat’s relative distance from the stimulus with scores ranging from 0, indicating that a rat spent the whole session on top of the stimulus, to 1 in which case a rat spent the whole test session furthest away from the stimulus in the opposite corner of the arena.

### 2.5 Data analysis

Data were analysed in R. 3.0.2 [61]. We used linear mixed models (LMM) using the Lme4 package to investigate the rats’ behavioural responses across the three risk contexts. We ran four LMMs with time spent out of cover, time in head-out, time hidden and relative distance to the stimulus as response variables. In each model, we included risk context (novel, predator odour, conditioned), sex, the interaction between them and body weight as fixed effects and rat identification (ID) was included as a random factor to account for the repeated-measures structure of our data. To minimize the number of analyses, we investigated sex differences in each context using the initial LMMs and the multcomp package (d.f.=58) [62]. Models were run for each of the behavioural responses separately to determine sex differences across the different contexts in detail. To determine habituation effects in the novel context, we ran an LMM with activity as response variable, sex, time (5 min time points), and the interaction between them as fixed effects, and rat ID as a random factor; *t*-tests were used to compare the activity of males during the first 10 min and last 10 min of the novel context session. To determine whether males and females also differed in how they dealt with the potential threat of the predator over time, we ran two additional LMMs for the predator odour context: we split the behavioural recordings in four 5 min sections and used the time spent hidden and head-out in each time section as response variables, time section (0–5 min, 5–10 min, 10–15 min, 15–20 min), sex and the interaction between them as fixed effects, and rat ID as a random factor. We only ran these models for the predator odour context as we expected the most pronounced differences in risk-assessment and hiding behaviour in this context. Finally, to better understand how the associatively learned responses of males and females were related to their behaviour when exposed to the predator odour, we ran planned contrasts using the four initial LMMs to compare males’ and females’ behavioural changes from the predator odour context to the conditioned context. Minimal adequate models were obtained by backward stepwise elimination and statistics for non-significant terms were obtained by adding each non-significant term to the minimal model using maximized log-likelihood. The residuals for all models were inspected to ensure normality, linearity and homogeneity of variance. In addition, we investigated whether the time individuals spent out of cover or in head-out during the second session could explain the time individuals spent out of cover during the third session using Spearman rank correlation tests. Owing to a technical problem, the predator odour trial of one female had to be excluded. As weight did not have a significant effect on any of the measured behaviours and was not the focus of the study, its effects are not further described below. All results with 0.10≥*p*>0.05 are reported as trends and *p*≤0.05 as significant. Means are quoted ± s.e. throughout.

## 3. Results

### 3.1 Sex differences in the novel context

**Figure 1.**
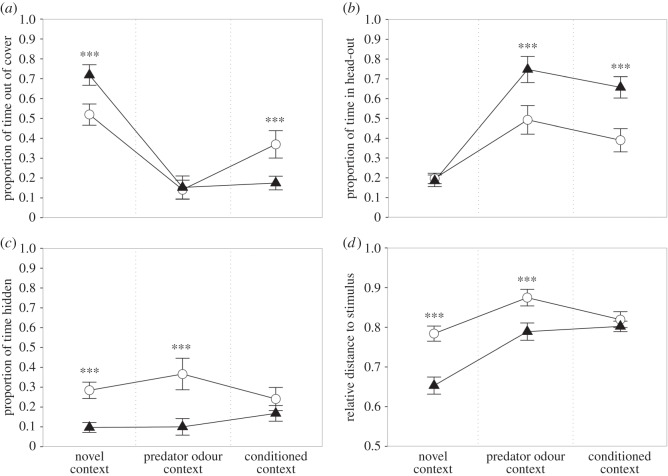
The proportion of time that males (*n*=30; triangles) and females (*n*=29; circles) spent (*a*) out of cover, (*b*) in head-out, and (*c*) hidden in the hide-box, as well as (*d*) their relative distance to the stimulus during the novel context, the predator odour context and the conditioned context. Data are presented as *means*±*s*.*e*. Significant sex differences for each context are indicated with *p<0.05, **p<0.01 and ***p<0.001.

When first placed in the open field test arena, i.e. the ‘novel context’, males spent more time out of cover than females (*z*=3.74, *p*<0.001; figure 1*a*), spent the same time in head-out (*z*=−0.22, *p*=0.994; figure 1*b*) and were less time hidden (*z*=−3.74, *p*<0.001; figure 1*c*). Males were on average closer to the control stimulus than females (*z*=6.68, *p*<0.001; figure 1*d*). Both sexes became less active over time, following a pattern with a polynomial curve that levelled off towards the end of the session ($\chi_{2}^{2}=44.81$; *p*<0.001; electronic supplementary material, figure S2), with females decreasing their activity more than males early on in the trial but having similar activity as males towards the end of the session (sex×time: $\chi_{2}^{2}=10.69$; *p*=0.005). Although females were slightly more active during the first half of the trial (*t*_118_=2.50, *p*=0.014; *r*^2^=0.04), males and females had the same activity level during the latter half of the trial (*t*_118_=0.45, *p*=0.652; *r*^2^=0.00).

### 3.2 Sex differences in the predator odour context

**Figure 2.**
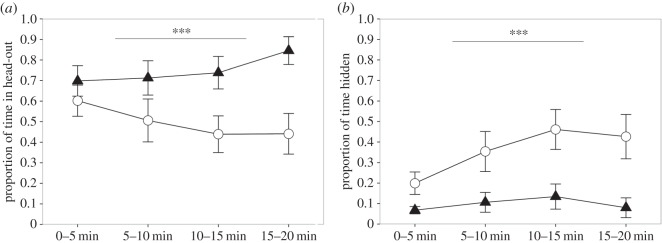
The proportion of time that males (*n*=30; triangles) and females (*n*=29; circles) were in (*a*) head-out and (*b*) hidden in the hide-box for each of four 5 min sections of the 20 min predator odour context session. Data are presented as means±*s*.*e*. Significant overall sex×time effects are indicated with *p<0.05, **p<0.01 and ***p<0.001.

Upon exposure to the field test arena containing the cat odour stimulus, males and females spent the same amount of time out of cover (*z*=0.23, *p*=0.993; figure 1*a*). However, males showed considerably more head-out behaviour (*z*=4.74, *p*<0.001; figure 1*b*), were less time hidden in the hide-box (*z*=−5.33, *p*<0.001; figure 1*c*), and were on average closer to the stimulus (*z*=4.41, *p*<0.001; figure 1*d*) than females. There were also sex differences in the rats’ behavioural responses over time: relative to females, males showed an increase in time spent in head-out across the session ($\chi_{3}^{2}=17.11$; *p*<0.001; figure 2*a*), while females relative to males spent increasingly more time hidden in the hide-box ($\chi_{3}^{2}=16.79$; *p* < 0.001; figure 2*b*).

### 3.3 Sex differences in the conditioned context

In the conditioned context, i.e. when the cat odour stimulus had been removed, males spent on average less time out of cover than females (*z*=−3.66, *p*=0.001; figure 1*a*), more time in head-out (*z*=4.99, *p*<0.001; figure 1*b*), and the same time hidden in the hide-box (*z*=−1.45, *p*=0.374; figure 1*c*). The average distance to the stimulus was not significantly different between males and females (*z*=0.87, *p*=0.743; figure 1*d*). Looking at the change in behaviour from the predator odour context to the conditioned context, females increased their time spent out of cover relatively more than males (*t*_115_=−3.48, *p*<0.001, *r*=0.31), while males and females showed the same decrease in time spent in head-out (*t*_115_=0.16, *p*=0.872, *r*=0.02). Furthermore, while males increased their time spent hiding, females decreased their time hidden in the hide-box (*t*_115_=3.08, *p*=0.003, *r*=0.28). Females also moved closer towards the stimulus (*t*_115_=−3.13, *p*=0.002, *r*=0.28). The time individuals spent out of cover during the conditioned context was positively correlated with the time they spent out of cover when exposed to the predator odour (males: *r*_*s*_=0.58, *p*<0.001; females: *r*_*s*_=0.78, *p*<0.001) but not to their time spent in head-out in that context (males: *r*_*s*_=−0.30, *p*=0.108; females: *r*_*s*_=0.01, *p*=0.972).

## 4. Discussion

To understand how males and females differ in their behavioural responses to risk, we measured rats’ behaviour across three different contexts in an open field test containing cover. We found that in the mild risk situation when the environment was novel, males spent more time out of cover and hid less than females. When exposed to the high-risk context containing the odour of a cat, both sexes increased their time in the hide-box considerably, but males spent significantly more time in head-out while females spent more time hidden completely. Finally, after being subjected to the predator odour, rats were re-exposed to the same context but without the cat odour. In this third condition, both sexes showed strong conditioned responses, with increased hiding and risk-assessment behaviour relative to their first exposure to the novel context, but this effect was stronger in males, and now females spent more time out of cover. These results suggest that male and female rats differ in how they cope with risk, both in terms of their direct behavioural responses (novel and predator odour contexts) and their learned responses (conditioned context).

Our findings that males explored more in the novel context and were closer to the stimulus and hid less than females in both the novel and predator odour contexts suggest that males are more risk-prone than females. This is in line with most other studies using a range of species [7,9,10,22] but contrasts with several rodent studies that report females are more exploratory [29,33,63]. Most of the latter studies however used tests that did not offer a place to hide [64], which contrasts with how most animals live in the wild [50,57]. As rats are fossorial and have a natural response to threat by hiding or running away [36,50], such tests that lack cover are less likely to measure voluntary exploration [65]. This may help explain the contrasting findings and interpretation of such studies [19,33] compared to those that used predatory threat and offered a place to hide [47,66]. Although we did not observe any sex differences in the time spent out of cover in the predator odour context, this may be owing to a ceiling effect, also documented by others [60], as both males and females showed very strong responses and spent the majority of their time in the hide-box [54,67,68]. Interestingly, while females spent increasingly more time hidden in the hide-box during the predator odour session, males spent more time in head-out [20,69] and increased this behaviour during this test session. Coming partly out of cover (head-out) enables the gathering of some information about the possible presence of the predator [70]. However, it is a relatively more risky strategy than staying concealed completely, especially in the high-risk context of predator odour, which suggests that a predator may be, or has recently been, nearby. Therefore, although females may generally be expected to show more sampling behaviour than males [41,45], this behaviour may be suppressed in more risky situations if hiding options are available. This is supported by the findings of a study that repeatedly exposed rats to cat odour and found that females only started to display high levels of head-out behaviour after repeated exposures, when the risk had relatively diminished [71]. These findings highlight that it is important to not only consider the magnitude of the behavioural response to a stressor, such as time spent hiding, but also the type of behavioural response, such as the time spent hiding completely versus sampling the environment by coming partly out of cover, see also [72].

By exposing rats to the same environment in which they previously experienced predator odour, we aimed to investigate whether males and females show different associatively learned responses to risk. The finding that both sexes still spent the majority of their time in the hide-box avoiding the stimulus, even though the cat odour had been removed, suggests strong conditioning effects in both males and females, conforming to previous studies [47,52,54,56,60]. However, we also found that, in contrast to the other test contexts, females in the conditioned context spent more time out of cover and decreased their distance to the stimulus while males did not. Based on the finding that males took more risk in the other two contexts, this finding suggests that males have stronger conditioned avoidance behaviour, which suggests that males are more affected by their former experiences than females, or alternatively, that females did not form as strong an association between the cat odour and the open field test. The time individuals spent out of cover in the conditioned context was not correlated to risk-assessment behaviour during the predator odour context. In other words, males did not show stronger conditioning because of potentially having gathered more information in the previous test context. The relative increase in exploratory behaviour in the conditioned context by females compared to males may be explained by females trying to update their information about the state of the changed environment [31,45,73,74], supporting the idea that females have a more reactive response. This is in line with studies showing that females typically sample more, are more responsive, and are more readily influenced by available contingencies [41,43,45,46]. That males may typically act more on the basis of previous experiences while females may rely more on a detailed and up-to-date account of their environment may help explain why males are generally faster at making decisions and learning discrimination tasks [38,40,41,75]: males may choose options with potentially large rewards more quickly and stick to them, while females keep on sampling their environment [39,41]. It is interesting to note that similar effects as we describe here at the level of the sexes have been documented extensively at the individual level (‘coping styles’) [72,76]. An exciting area for future research is therefore to explore sex differences in the context of coping styles, and in particular, to focus on the relationship between risk-taking behaviour and the behavioural and physiological stress-response in males and females [48].

The arena we used was conforming to previous work investigating direct and conditioning effects of predator odour [51,53,55,56], but is not standard for investigating open field behaviour. Although qualitatively the same responses may be expected in a smaller environment [64,77], a larger environment would have been needed to fully determine how males and females respond to a novel environment and may compromise direct comparisons to the results of other open field studies. Future research should investigate aspects of risk-related behaviour in larger, more natural environments. It may be suggested that the observed sex differences in the predator and conditioned contexts are attributable to a sex difference in habituation to the novel field test. Although no control groups were tested for habituation effects in the arena without the odour stimulus for the three sessions, general and sex-specific habituation effects are unlikely to have played a role [78]. First of all, our experimental paradigm was based on that used by a range of studies to investigate cat odour effects [51,53,55,56]. These studies used a similar-length session in a similarly sized arena to our ‘novel context’ session to control for any habituation effects. Second, close inspection and analysis of our rats’ activity patterns during the novel context trials shows a clear drop in activity that levels off towards the end of the session (see the electronic supplementary material, figure S2), confirmed by statistical analyses and which fits the pattern of an habituation curve. Although females were significantly more active during the first 10 min of the trial, both males and females had similar activity levels in the final half of the novel context trial. Therefore, sex differences in the predator odour context and conditioned context are unlikely to be owing to a sex difference in habituation, and additional control groups that are repeatedly tested in an empty arena not warranted in compliance with the principles of the 3Rs [79]. Future work is needed to properly investigate the relationship between habituation and risk-taking in males and females. Finally, it may be suggested that the observed sex differences in our study are due to the females’ oestrous cycle. However, although no data could be collected on the females’ oestrous cycle, it is unlikely to have affected our results. Not only have other studies documented that the oestrous cycle did not significantly affect female rat behaviours [80,81], Norway rats do not seem to synchronize their oestrous cycle [82] and we observed no sex differences in variability over time across the consecutive tests (see also [83]). Nevertheless, future studies may help understand how changes in oestrous cycle may potentially affect risk-taking and related association learning over time.

While previous studies have shown that sex differences exist in risk-taking [7,9,10,20–23] and learning [19,31,38,39], here we show a link between them, with males being more risk-prone and relying more on former experiences, and females being more sensitive to their environment and showing more up-to-date information acquisition. Such a link between risk-taking and learning may be explained by underlying risk–reward trade-offs [49]. In the same way that it is safer to hide than to explore in a risky context, it might be safer to guide decisions by a slower, more detailed acquisition and assessment of information, and to update this information whenever the situation changes. Such sex differences in risk-taking and associatively learned responses may ultimately be explained by differences in reproductive investment and resulting different fitness expectations for males and females [57,59]. Furthermore, the higher costs and potentially increased predation risk that comes with the higher reproductive investment of females may make an active hiding response more important [36]. Similar effects may therefore be expected for other species in which females have a higher reproductive investment, and contrasting effects for species in which males have a higher reproductive investment. A great example of this is the pipefish, a species in which males invest more in their brood than females, and females show more risk-prone behaviour [23]. To further our understanding of these broad patterns of behavioural variability, it would be interesting to investigate the link between risk-taking and learning style in species with large, small and opposite sex differences in reproductive investment. In the context of observed sex effects, it is interesting to highlight that recently a conceptual framework has been developed with supporting evidence that links personality types to differences in the way individuals process, store or act on information (termed their ‘cognitive style’) [49,84]. For example, bolder, more exploratory, proactive animals tend to be faster at learning initial discriminations [75,85,86], but rely more on former experience [87] and make more errors in tests of reversal learning [88], while, in contrast, shy, slow exploring and reactive animals are more sensitive to environmental stimuli, more flexible in their behaviour, and better at adapting to changes in an already learned task [72,88]. An exciting area for future research would therefore be to investigate to what extent such effects at the individual level might be related to the observed effects at the level of the sexes.

To conclude, by investigating the behavioural responses of male and female rats in contexts with low, high and conditioned risk, we show that males and females differ in how they cope with risk in terms of risk-taking and learning style. Males are more risk-prone and respond more strongly to previous experiences, while females are more risk-averse and respond more to their current environment. A further integration between the behavioural ecological and biomedical sciences may help to determine both the proximate neuro-, physiological and endo-neurocrinological causes, as well as ultimate consequences of these broad patterns of behavioural variation.

## Supplementary Material

Figure S1: Schematic representation of the novel field test arena. Figure S2: Habituation curves for males and females for the novel open field test session.

## Supplementary Material

Table S1: Individual behavioural data for all male and female subjects for the three different contexts.

## Data accessibility

The dataset supporting this article has been uploaded as the electronic supplementary material.
