# Supplementary material for: Sex differences in risk-taking and associative learning in rats
Source: R Soc Open Sci. 2015 Nov 4;2(11):150485. doi: 10.1098/rsos.150485 (PMC4680619; doi:10.1098/rsos.150485)
Supplement: Figure S1: Schematic representation of the novel field test arena. Figure S2: Habituation curves for males and females for the novel open field test session. [file rsos150485supp1.doc]

**Sex differences in risk-taking and associative learning in rats**

Jolle W. Jolles^1*^, Neeltje J. Boogert^1^, Ruud van den Bos^2^

*1 Department of Zoology, University of Cambridge, Downing Street, Cambridge, UK, CB2 3EJ*

*2 Department of Organismal Animal Physiology, Faculty of Science, Radboud University Nijmegen, Nijmegen, Netherlands*

**Figure S1**

**
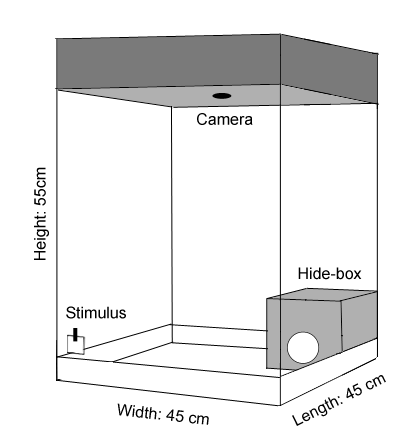
**

Schematic representation of the novel field test arena, containing a hide-box in one corner and a control or predator odour stimulus in the opposite corner.

**Figure S2**

**
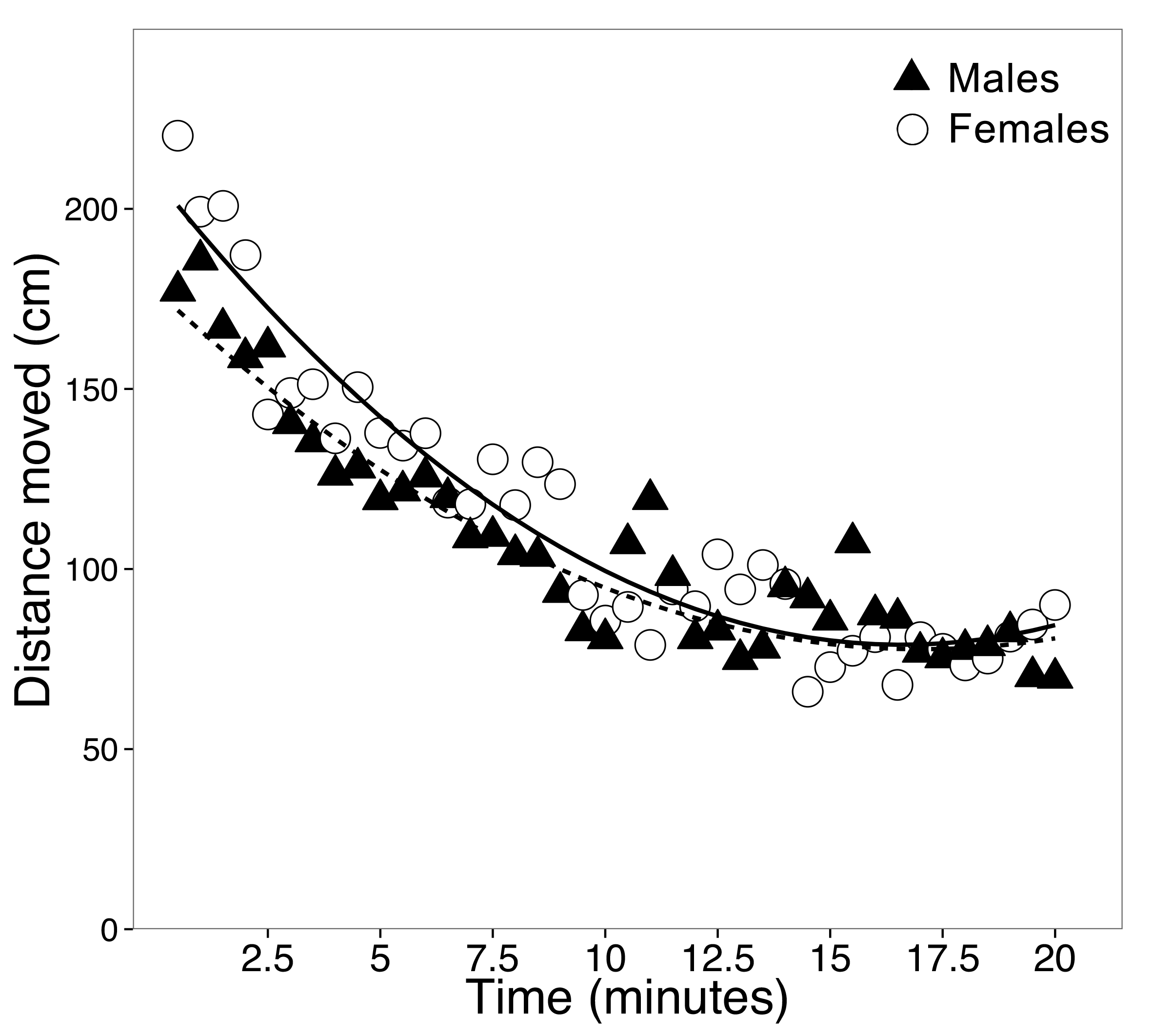
**

The distance moved by males (*N* = 30) and females (*N* = 30) during the 20-min novel context trial. Data is based on trajectory data of the individual rats, with each point representing the mean of all 30 individuals of one sex at 30-second time points. A clear reduction in movement over time was apparent for both sexes, which fits the pattern of the classical habituation curve and a lack of change in activity towards the end of the trial for both males and females (dashed and solid line respectively, based on a LMM; see results).
